# Evaluation of Mental Workload among ICU Ward's Nurses

**DOI:** 10.15171/hpp.2015.033

**Published:** 2016-01-30

**Authors:** Mohsen Mohammadi, Adel Mazloumi, Zeinab Kazemi, Hojat Zeraati

**Affiliations:** ^1^Department of Occupational Health Engineering, Tehran University of Medical Sciences, Tehran, Iran; ^2^Department of Epidemiology and Biostatistics, Tehran University of Medical Sciences, Tehran, Iran

**Keywords:** Intensive care units, Mental workload, NASA-TLX, Nurses, Performance obstacles

## Abstract

**Background:** High level of workload has been identified among stressors of nurses in intensive care units (ICUs). The present study investigated nursing workload and identified its influencing perfor­mance obstacles in ICUs.

**Methods:** This cross-sectional study was conducted, in 2013, on 81 nurses working in ICUs in Imam Khomeini Hospital in Tehran, Iran. NASA-TLX was applied for assessment of workload. Moreover, ICUs Performance Obstacles Questionnaire was used to identify performance obstacles associated with ICU nursing.

**Results:** Physical demand (mean=84.17) was perceived as the most important dimensions of workload by nurses. The most critical performance obstacles affecting workload included: difficulty in finding a place to sit down, hectic workplace, disorganized workplace, poor-conditioned equipment, waiting for using a piece of equipment, spending much time seeking for supplies in the central stock, poor quality of medical materials, delay in getting medications, unpredicted problems, disorganized central stock, outpatient surgery, spending much time dealing with family needs, late, inadequate, and useless help from nurse assistants, and ineffective morning rounds (P-value<0.05).

**Conclusion:** Various performance obstacles are correlated with nurses' workload, affirms the significance of nursing work system characteristics. Interventions are recommended based on the results of this study in the work settings of nurses in ICUs.

## Introduction

Excessive workload has been identified as a significant stressor across different occupations.^1^ Jobs with high level of workload and occupations with inappropriate work schedule would diminish operators' performance and results in memory impairments, irritability, and reduced learning capacity.^2,3^ Regulating task demands in a way that prevents individuals from being under load or overload has considerable importance to ensure their safety, health, comfort, and productivity.^4^

Nurses’ work in complex environments with high technology results to increase the amount of workload they are exposed to.^2^ Nurses in the intensive care units (ICUs) have extensive responsibilities and limited latitude, which expose them to an extremely high workload,^5^ both physically and mentally.^6^ Nurses must continuously cope with the requests of patients and their families, and unwantedly involve in the strong emotional issues related to patients.^7^ Moreover, they are involved in multiple decision-makings in urgent situations vital to patients' lives.^5,8,9^ Abbey et. al. reported 3081 activities undertaken by ICU nurses during the day shift, of which 43% were performed simultaneously. This result implies the risk of medical errors and the probability of reduction in patients’ safety.^10^ Nursing workload is entified as an important contributor of patients' safety and quality of care in ICUs.^7,11,12^ Beckmann et. al. investigated the problems associated with nursing staff shortage in ICUs in Australia, and reported that nursing shortages would increase the rate of incidents and decrease the patients' safety and quality of care.^13^ High level of workload and the staff/patient ratio were highly correlated with patients' mortality.^14^

Measuring nursing workload would have positive influences on the management of nursing workload and consequently on the provision of safety and quality of patients' care.^15^ Carayon and Gürses classified the nurses' workload measures into four groups as follows: 1) workload measures at the unit level, 2) workload at the job level, 3) workload at the patient level, and 4) workload at the situation level.^7^

Situation-level investigates the nursing workload from a micro-level approach, for instance design characteristics of the clinical micro system, a specific event, or even workload over a period.^7^ The workload-developed measures in the field of human factors can be applied for measuring workload at the situation-level.^7^ The ICU work system can be considered as a clinical micro system that could be investigated in order to identify the contributory factors in nursing workload.^16^ The nurses' workload can be negatively affected by factors related to their work system, called performance obstacles. Gürses and Carayon defined performance obstacles as "the work factors in the immediate work setting of ICU nurses that increase their workload beyond what is expected.^9^

According to the above-mentioned statements related to workload and its effects on the quality of care and patients' safety, and inspiring relevant researches^7,9,16^ the present study aimed to investigate nursing workload and those performance obstacles that increase the workload in ICUs, in one of Tehran University of Medical Sciences hospitals. The performance obstacles of ICU nurses, in Iran, have not been delineated in previous researches.

## Materials and Methods

The present cross-sectional study was done in 2013 on 81 nurses working in ICUs of Imam Khomeini Hospital in Tehran, Iran, affiliated to Tehran University of Medical Sciences. Informed consent forms were signed by all volunteered participants. Furthermore, the study was approved by the Ethics Committee of Tehran University of Medical Sciences. The following tools were used to collect data.

*NASA Task Load Index:* NASA-TLX is one of the well-known subjective workload assessment tools, presented by Hart and Staveland.^17^ This is a multidimensional instrument, which gives a total score according to six subscales including: mental demand, physical demand, temporal demand, performance, effort, and frustration.^18^ The calculation of this scale was done according to the method presented earlier^19,20^ in which participants rate the level of their workload for each subscale on a 10-cm visual-analog scale and then these scores are altered to a 0-100 scale. Consequently, two scores can be calculated consisting of Raw-TLX, which is the arithmetic average of the six scores, and Adaptive Weighted Workload (AWWL).The validity and reliability of this scale have been previously confirmed.^4^ A backward translation method was used for determining the face validity of the questionnaire and Cronbach’s alpha was calculated for determining its reliability.

*Questionnaire of Performance Obstacles of ICUs Nurses:* Questionnaire developed by Gürses and Carayon,^16^was used to identify performance obstacles associated with ICU nursing. In the first stage, cross-cultural adaptation of the questionnaire was performed by conducting semi-structured interviews with 15 nurses from ICUs, using a guide. The interview guide consisted of two open questions, designed to focus the interviews on associated performance obstacles. The interviews were done during shift hours, recorded and transcribed.

In the second stage, the stated performance obstacles were classified based on the qualitative model developed in a previous research,^16^ into ten groups including: physical work environment, tools and equipment, materials and supplies, inter-provider communication, information, intra-hospital transport of patients, patient-related factors, family-related factors, help from other personnel, and teaching institution. Finally, a questionnaire, consisting of 53 questions, was developed according to the identified obstacles.

The accuracy, relevancy, and comprehensiveness of the questionnaire were evaluated through asking 10^21^ managers and nurses in ICUs. In this stage, participants were asked to rate the relevancy, clarity, and comprehensiveness of each individual question. The relevancy was evaluated by four items (1=not relevant, 2=somewhat relevant but needs further revision, 3=relevant but needs minor revision, and 4=very relevant). The rate of clarity and comprehensiveness degree of each question was evaluated as well. The comments of the nurses were written during answering the questionnaire. The finalized questionnaire was filled out by 10 nurses twice with a two-week interval. The correlation coefficients between these two sets of scores were calculated for determining the reliability.

### Ethical Issues

This study was approved by the Ethics Committee of Tehran University of Medical Sciences.

### Statistical analysis

Statistical analysis was done using SPSS software, version 18. Descriptive statistics were used to describe the characteristics of the study variables. In order to investigate the difference of workload among people with different demographic variables One-Way ANOVA and T-test statistical tests were used. The relationships between performance obstacles and workload were determined using Spearman correlation coefficient. Furthermore, a multiple regression was run to predict the most important performance obstacles which influence workload.

## Results

A high percentage of participants rated relevancy and clarity of items within the performance obstacles questionnaire as relevant and very relevant, and clear and very clear, respectively. Moreover, totally, 60% of nurses assessed the comprehensiveness of the instrument as very comprehensive, 30% as comprehensive, and 10% as somehow comprehensive. The total relevancy and clarity of the questionnaire was obtained 97% and 96%, respectively (Table 1).

Regarding the reliability of the two questionnaires, Cronbach’s alpha coefficient was estimated 0.847 for NASA-TLX and 0.875 for performance obstacles questionnaire.

In addition, Spearman correlation coefficient was obtained 0.746 for two sets of scores related to performance obstacles questionnaire.

**Table 1:** Relevancy and clarity of performance obstacles categories

**Main categories**	**Relevancy**					**Clarity**				
	Frequency*				**Relevancy**	Frequency**				**Clarity**
	**1**	**2**	**3**	**4**	**(%)**	**1**	**2**	**3**	**4**	**(%)**
Physical work environment	0	0	3	7	100	0	0	2	8	100
Tools and equipment	0	0	5	5	100	0	0	1	9	100
Materials and supplies	0	0	0	10	100	0	0	2	8	100
Inter-provider communication	0	1	2	7	90	0	2	4	4	80
Information	0	0	0	10	100	0	0	2	8	100
Intra-hospital transport of patients	0	0	0	10	100	0	0	0	10	100
Patient related factors	0	2	4	4	80	0	2	2	6	80
Factors related to patients' family	0	0	0	10	100	0	0	0	10	100
Help from other personnel	0	0	0	10	100	0	0	0	10	100
Academic hospital	0	0	0	10	100	0	0	0	10	100
Total relevancy of the instrument					97					96

* 1=not relevant, 2=somewhat relevant but needs further revision, 3=relevant but needs minor revision, and 4=very relevant ** 1=not clear, 2=somewhat clear but needs further revision, 3=clear but needs minor revision, and 4=very clear

The mean age of ICUs nurses was 33.72(SD 5.54) and the mean job tenure was 6.45(SD 4.8) years. Descriptive data related to overall workload score and its subscales are presented in Table 2. As can be seen, physical demand (mean=84.17) was rated high, and frustration dimension (mean=54.49) was perceived as the least important by nurses. Statistical analyses were done to determine the demographic variables, which affect the workload of ICU nurses. Accordingly, among the demographic variables, age, job tenure, and education were significantly related to at least one of the NASA-TLX subscales (Table 3).

**Table 2:** Descriptive statistics related to workload and its subscales

**Workload variables**	**Mean**	**SD**	**Minimum**	**Maximum**
Mental demand	76	18.859	0	100
Physical demand	84.17	16.955	32	100
Temporal demand	76.46	19.849	30	100
Effort	81.40	15.244	42	100
Performance	78.57	17.364	21	100
Frustration	54.49	31.827	0	100
RTLX	75.11	12.248	45	95
AWWL	82.62	10.391	59	97

**Fig. 1:**
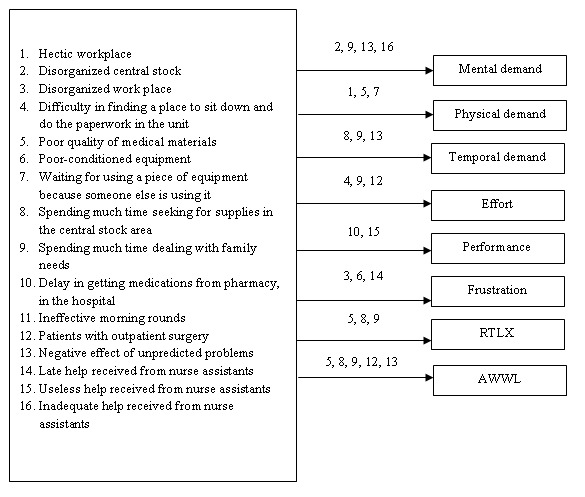
Summary of regression analysis to predict perfor-mance obstacles affecting workload

Spearman correlation coefficient was employed for determining the relationship between items of performance obstacles and workload. Table 4 represents those obstacles shown to have significant correlation with workload. Twenty-nine of the 53 performance obstacles were significantly correlated with at least one of the workload subscales, of which nine obstacles correlated with AWWL: hectic workplace, spending much time seeking for supplies in the central stock area, poor quality of medical materials, negative effect of unpredicted problems, patients with outpatient surgery, inadequate information from physicians about the patient(s), spending much time dealing with family needs, late help received from nurse assistants, negative effect of working in an academic hospital on the patients' care. A multiple regression was run to predict the most important performance obstacles affecting workload (Table 5). A summary of the results of regression analysis are presented in Figure 1.

**Table 3:** Statistical analysis results for workload subscales by demographic variables

Variables	*P*-value							
	**Mental Demand**	**physical Demand**	**Temporal Demand**	**Effort**	**Performance**	**Frustration**	**RTLX**	**AWWL**
Age (yr)^†^	0.082	0.265	0.295	0.006^*^	0.114	0.342	0.140	0.030^*^
Gender^‡^	0.432	0.653	0.473	0.664	0.669	0.457	0.198	0.453
BMI^†^	0.191	0.451	0.217	0.058	0.324	0.870	0.259	0.140
Job tenure ^†^	0.615	0.000^*^	0.001^*^	0.000^*^	0.108	0.325	0.006^*^	0.000^*^
Education^†^	0.270	0.656	0.520	0.290	0.000^*^	0.008^*^	0.161	0.671
Marital status ^†^	0.907	0.509	0.299	0.378	0.345	0.722	0.384	0.525

* RTLX=Raw Task Load Index, AWWL=Adaptive weighted Workload /† One Way ANOVA statistical /‡ T-test

Accordingly, 16 obstacles remained as the predictors of total workload and its subscales including:

-Physical work environments (difficulty in finding a place to sit down and do the paperwork, hectic workplace, and disorganized workplace); Tools and equipment (poor-conditioned equipment, waiting for using a piece of equipment because someone else is using it, spending much time seeking for supplies in the central stock area); Materials and supplies (poor quality of medical materials, delay in getting medications from pharmacy in the hospital, negative effects of unpredicted problems, and disorganized central stock); Patient related factors (patients with out-patient surgery); Factors related to patients family (spending much time dealing with family needs); Help from other personnel (late, inadequate, and useless help received from nurse assistants) and academic hospital (ineffective morning rounds).

**Table 4:** The association between performance obstacles and workload subscales, using Spearman correlation

**Performance obstacles**	***P*** **-value**							
	**MD**	**PD**	**TD**	**EF**	**PE**	**FR**	**RTLX**	**AWWL**
- Difficulty in finding a place to sit down and do the paperwork in the unit	0.432	0.017‏*	0.196	0.016‏*	0.952	0.688	0.085	0.067
- Crowded workplace	0.106	0.014‏*	0.265	0.509	0.138	0.505	0.071	0.116
- Hectic workplace	0.259	0.000‏*	0.082	0.218	0.701	0.631	‏*0.022	‏*0.035
- Disorganized work place	0.817	0.479	0.995	0.811	0.398	0.000‏*	0.062	0.233
- Poor climate condition of workplace	0.091	0.426	0.081	0.446	0.018‏*	0.179	0.508	0.364
- Disorganized patient rooms	0.351	0.737	0.654	0.207	0.795	‏*0.000	‏*0.010	0.071
- Using poor-conditioned equipment	0.925	0.171	0.697	0.391	0.951	0.001‏*	0.074	0.284
- Spending much time looking for equipment	0.480	0.408	0.496	0.090	0.067	‏*0.000	‏*0.020	0.197
- Wrong location of equipment	0.953	0.966	0.077	0.932	0.035‏*	0.252	0.483	0.471
- Waiting for using a piece of equipment because someone else is using it	0.593	0.043‏*	0.679	0.397	0.348	0.102	0.506	0.212
- Spending much time seeking for supplies in the central stock area	0.022‏*	0.064	0.003‏*	0.262	0.070	‏*0.000	‏*0.002	‏*0.012
- Not well-stocked non-isolation room	0.209	0.006‏*	0.325	0.449	0.630	0.087	0.060	0.157
- Poor quality of medical materials	0.285	0.001‏*	0.356	0.032‏*	0.248	0.495	‏*0.047	‏*0.038
- Delay in getting medications from pharmacy, in the hospital	0.450	0.342	0.815	0.916	0.000‏*	0.148	0.192	0.216
- Delay in getting medications from pharmacy, out of the hospital	0.348	0.738	0.647	0.525	0.004‏*	0.656	0.119	0.142
- Negative effect of unpredicted problems	0.010‏*	0.059	0.001‏*	0.600	0.137	0.485	0.322	0.006‏*
- Disorganized central stock	0.009‏*	0.486	0.953	0.493	0.116	‏*0.000	‏*0.014	0.135
- Patients with outpatient surgery	0.214	0.016‏*	0.078	0.012‏*	0.580	0.033‏*	0.250	0.039‏*
- Unnecessary detailed information related to patients given by the previous shift's nurse(s)	0.098	0.074	0.989	0.168	0.039‏*	0.205	0.864	0.788
- Nurses' inadequate communication with physicians	0.030‏*	0.836	0.889	0.992	0.456	0.027‏*	0.080	0.116
- Inadequate information from physicians about the patient(s)	0.010‏*	0.001‏*	0.343	0126	0.213	0.792	0.118	0.030‏*
- Spending much time dealing with family needs	‏*0.000	‏*0.005	‏*0.004	‏*0.012	0.488	0.202	‏*0.000	‏*0.000
- Receiving many phone calls from family members	0.611	‏*0.013	0.615	0.455	0.584	0.471	0.616	0.506
- Late help received from nurse assistants	‏*0.003	0.945	0.454	0.507	0.089	‏*0.000	‏*0.007	‏*0.046
- Inadequate help received from nurse assistants	‏*0.000	0.688	0.977	0.344	0.066	0.070	0.147	0.213
- Useless help received from nurse assistants	0.004‏*	0.181	0.400	0.426	0.000‏*	0.061	0.945	0.782
- Ineffective morning rounds	0.622	0.001‏*	0.264	0.140	0.158	0.488	0.589	0.122
- Delay in receiving new medical orders for patients	0.166	0.576	0.219	0.667	0.941	0.896	0.146	0.155
- Negative effect of an academic hospital on the patients' care	0.212	0.002‏*	0.147	0.301	0.855	0.282	‏*0.024	‏*0.042

* MD=Mental Demand, PD=physical Demand, TD=Temporal Demand, EF=Effort, PE=Performance, FR=Frustration, RTLX=Raw Task Load Index, AWWL=Adaptive weighted Workload

**Table 5:** Multiple linear regression analysis results for workload scores by performance obstacles

**Workload subscales**	**Performance obstacles**	**Unstandardized** ** Coefficients**		**Standardized Coefficients**	***P*** **-value**
		**B** ^*^	**Std. Error**	**Beta**	
Mental	Constant	101.885	5.456		0.000
	Inadequate help received from nurse assistants	-4.660	1.187	-.364	0.000
	Spending much time dealing with family needs	-15.355	4.560	-.309	0.001
	Negative effects of unpredicted problems	25.036	8.846	.253	0.006
	Disorganized central stock	-4.526	1.616	-.254	0.007
Physical	Constant	81.518	5.145		0.000
	Hectic workplace	-3.281	1.458	-.215	0.027
	Poor quality of medical materials	-8.892	3.076	-.263	0.005
	Waiting for using a piece of equipment because someone else is using it	8.437	3.086	.246	0.008
Temporal	Constant	86.739	3.592		0.000
	Spending much time dealing with family needs	-13.931	5.289	-.267	0.010
	Negative effects of unpredicted problems	25.875	8.063	.316	0.002
	Spending much time seeking for supplies in the central stock area	-13.228	4.493	-.302	0.004
Effort	Constant	92.142	3.184		0.000
	Spending much time dealing with family needs	-12.679	4.009	-.316	0.002
	Patients with outpatient surgery	-7.161	3.137	-.232	0.025
	Difficulty in finding a place to sit down	-7.487	3.563	-.213	0.039
Performance	Constant	115.448	20.203		0.000
	Delay in getting medications from pharmacy in the hospital	8.947	3.774	.251	0.020
	Useless help received from nurse assistants	3.798	1.494	.265	0.013
Frustration	Constant	93.281	8.614		0.000
	Late help received from nurse assistants	-7.488	2.080	-.360	0.001
	Poor-conditioned equipment	-14.567	6.590	-.229	0.030
	Disorganized workplace	-6.878	3.394	-.219	0.046
RTLX	Constant	86.140	2.504		0.000
	Spending much time dealing with family needs	-11.454	3.173	-.360	0.001
	Poor quality of medical materials	-7.709	2.336	-.314	0.001
	Spending much time seeking for supplies in the central stock area	-7.810	2.675	-.291	0.005
AWWL	Constant	93.012	2.181		0.000
	Spending much time dealing with family needs	-10.159	2.639	-.373	0.000
	Poor quality of medical materials	-7.335	1.974	-.349	0.000
	Patients with outpatient surgery	-4.863	1.996	-.225	0.017
	Spending much time seeking for supplies in the central stock area	-5.325	2.225	-.232	0.019
	Negative effects of unpredicted problems	11.656	5.127	.215	0.026

**Regression coefficient

## Discussion

In the present study, performance obstacles that affect situational mental workload of ICUs nurses were investigated based on conceptual workload model presented by Gürses, Carayon.^16^ An adapted version of the Performance Obstacles of ICUs Nurses questionnaire was developed, and its validity and reliability was determined. Furthermore, in this study, NASA-TLX, which is a reliable tool for assessing workload, was translated into Persian and employed for evaluating workload of nurses in ICUs. Nursing performance obstacles, which impact their workload, were categorized into 10 groups including: physical work environment, tools and equipment, materials and supplies, inter-provider communication, information, intra-hospital transport of patients, patient related factors, factors related to patients' family, help from other personnel, and academic hospital. This classification is consistent with those in the study by Gürses and Carayon.^9^ Peters et. al. also reported performance obstacles to be in eleven groups consisted of: job-related information, tools and equipment, supplies and materials, budgetary support, required services and help from others, task preparation, time availability, work environment, scheduling of activi-ties, transportation, and job-relevant authority.^22^ In addition, using observational methods, nurses experi-enced five types of problems including missing or incorrect information; missing or broken equipment; waiting for a (human or equipment) resource; missing or incorrect supplies; and simultaneous demands on their time^23^.

The categories identified in the mentioned studies^22,23^ provide good information related to per-formance obstacles of health care workers.

However, the classification by Gürses et. al. and in the present study is specifically related to ICU nursing.

Moreover, this classification seems to be comprehensive due to its system approach (macro ergonomics) and adoption from the Balance Theory of job design.^16,24,25^ Using this model in our study, we were capable to cover and investigate almost various aspects of work, which impact nurses’ performance in the intensive care units.

Regarding the nurses workload, physical demand was the most important dimensions of NASA-TLX by the nurses. NASA-TLX was the most reliable and valid questionnaire to measure workload in health care settings^26^. Moreover, our results showed that mental workload was the highest valued dimensions of NASA-TLX. Mental demand was also the most important dimensions of ICUs nurses.^22^ The discrepancy between the result of our study and the two mentioned studies might be explained by the differences in working conditions and technologies used by nurses in our study comparing with those used by nurses in other countries. As work environments be-come more complex and new technologies are used by health care workers, the mental demand of these occupational groups is increased.^27^ In this sense, present research revealed that hectic work place, poor quality of medical materials, and waiting for using a piece of equipment because someone else is using it were the three obstacles highly correlated with physical demand. This might be an explanation for the high level of physical demand among ICUs nurses.

Overall, regarding performance obstacles, ICUs nurses deal with a wide range of performance obstacles during their shift. Those common obstacles in our study and earlier,^22^ remained in the final model, included: difficulty in finding a place to sit down and do the paperwork, hectic workplace, disorganized workplace, poor-conditioned equipment, delay in getting medications from pharmacy, and spending much time dealing with family needs. Late, inadequate, and useless help received from nurse assistants were significantly correlated with the workload^22^. This shows the weakness in this aspect of work among ICUs nurses in the present study.

As for limitations, we relied solely on the subjective data for assessment of workload, which may be biased by nurses. Therefore, it is recommended to focus on objective methods in order to evaluate nurses’ workload in future studies. Moreover, high number of questions accompanied by nurses' busy schedule to fill it out is of the limitations of this study.

## Conclusion

Health professionals, especially nurses, work under a high stress condition. Therefore, identifying those causes, which affect nurses’ workload, is highly important. We investigated nurses’ workload based on performance obstacles model. The correlation between various performance obstacles and nurses' workload in the present study affirm the critical role of nursing work system characteristics, which should be taken into account while redesigning the work. Future projects in this area may include a comparison of performance obstacles of ICU nurses between private and public or academic and nonacademic hospitals. Using an objective tool for assessment of ICU nurses workload would also shed light for development of workload countermeasures.

## Acknowledgements

This study was a part of Master's thesis in Occu-pational Health Engineering supported by Tehran University of Medical Sciences. The authors declare that there is no conflict of interests.
